# Beneficial Effects of Marine Algal Compounds in Cosmeceuticals 

**DOI:** 10.3390/md11010146

**Published:** 2013-01-14

**Authors:** Noel Vinay Thomas, Se-Kwon Kim

**Affiliations:** 1 Marine Biochemistry Lab, Department of Chemistry, Pukyong National University, Busan 608-737, Korea; E-Mail: noelthomas@pknu.ac.kr; 2 Marine Bioprocess Research Center, Pukyong National University, Busan 608-737, Korea

**Keywords:** marine algae, sulfated polysaccharides, cosmeceuticals, phlorotannins, atopic dermatitis

## Abstract

The name “cosmeceuticals” is derived from “cosmetics and pharmaceuticals”, indicating that a specific product contains active ingredients. Marine algae have gained much importance in cosmeceutical product development due to their rich bioactive compounds. In the present review, marine algal compounds (phlorotannins, sulfated polysaccharides and tyrosinase inhibitors) have been discussed toward cosmeceutical application. In addition, atopic dermatitis and the possible role of matrix metalloproteinase (MMP) in skin-related diseases have been explored extensively for cosmeceutical products. The proper development of marine algae compounds will be helpful in cosmeceutical product development and in the development of the cosmeceutical industry.

## 1. Introduction

Marine macroalgae are taxonomically classified as algae, and they belong to four major seaweed classes, the rhodophyceae (red algae), the phaeophyceae (brown algae), the cyanophyceae (blue-green algae), and the chlorophyceae (green algae). The wide diversity in the biochemical composition of seaweeds provides an excellent choice to explore a variety of biologically active components in their bodily composition with a broad range of physiological and biochemical characteristics, many of which are rare or absent in other taxonomic groups [1,2,3,4,5,6,7,8,9,10,11]. Marine algae are a famous delicacy in some parts of Asia, and also a well-known source of important food phlorotannins [12], pigments [13] and sulfated polysaccharides [14,15,16,17]. Compared to the terrestrial plants and animal-based foods, seaweed is rich in some health-promoting molecules and materials such as, dietary fiber, ω-3 fatty acids, essential amino acids, and vitamins A, B, C, and E [18], which is essential for cosmeceutical product development [19]. In addition, marine algae are considered as sea vegetables not only for consumption, but also as an alternative medicine since ancient times [20] for skin-related diseases. In other words, the marine environment is many folds richer in its biodiversity, thereby making marine organisms and their metabolites unique [7]. The majority of the investigations on the metabolites derived from brown algae [21] have revealed their potential antioxidant [22], anti-inflammatory [22,23,24,25], antidiabetic [26], antitumor [27], antihypertensive [28], and anti-allergic [8] properties, as well as their role in hyaluronidase enzyme inhibition [29], neuroprotection [30], bone-related diseases [31,32,33] and in matrix metalloproteinase (MMPs) inhibition activity [29]. In addition, marine algae-derived compounds have been recently given much importance in cosmeceutical product development [34,35,36]. 

As many Asian females prefer a fairer skin tone, skin-whitening products have become, and continue to be, the best-selling skincare products in Asia [37]. Epidemiological and clinical studies have specified that consumption of plant-derived foods and drinks, such as tea, red wine, and soya bean products could reduce the risk of oxidative-damage-related diseases such as aging and other lifestyle diseases [38]. The marine environment is enriched with a variety of organisms that harbor a wide range of biologically important compounds that are useful for the cosmeceutical benefit of humans. Until now, only a few organisms have been exploited for the screening of cosmeceutical compounds from marine species. Presently, several studies have provided insight into biological activities of marine algae in promoting skin, health, and beauty products. Hence, marine algae have a great potential to be used for cosmeceutical application. In the present review, an attempt has been made to throw light on the cosmeceutically important ingredients—phlorotannins, sulfated polysaccharides and tyrosinase inhibitors—present in marine edible algae, by exploring and discussing them in regards to further industrial development.

## 2. Marine Algae in Cosmeceuticals

Cosmeceuticals have attracted increased attention because of their beneficial effects on human health. Bioactive substances derived from marine algae have diverse functional roles as a secondary metabolite, and these properties can be applied to the development of cosmeceuticals [19,39,40,41,42,43,44].

### 2.1. Skin Health Protection and Skin Whitening

Skin wrinkling is normally attributed by the reactive oxygen species (ROS) [45] which is caused by oxidative stress. ROS stimulates mitogen-activated protein kinases that phosphorylates transcription factor activator protein 1, which, in turn, results in upregulation of matrix metalloproteinase (MMPs) that contribute for the degradation of skin collagen, ultimately leading to skin aging [46,47]. The gelatinases, which include MMP-2 and MMP-9, promote UV-induced skin damage. It is reported that sun-damaged skin shows significantly elevated levels of active gelatinases (MMP-2 and -9) than intrinsically aged skin [48]. *In vitro* studies on methanol extract from marine alga *Corallina pilulifera* (CPM) have revealed that CPM has the ability to prevent UV-induced oxidative stress and also the expressions of MMP-2 and MMP-9 in human dermal fibroblast (HDF) cells. This clearly suggests the role of phenolic compounds from marine algae as potential MMP inhibitors [49]. As it is evident that unregulated expression of MMPs leads to photoaging, many research groups are emphasizing their research goals to check the ability of marine-derived phlorotannins as potential anti-photoaging agents [50,51,52,53,54,55]. Moreover, the ROS that include hydrogen peroxide, hydroxyl radical and superoxide anion are involved in metabolic diseases, especially chronic inflammation. In chronic inflammation, pro-inflammatory cytokines induce MMPs that degrade the extracellular matrix and contribute to several inflammatory disorders. 

**Figure 1:**
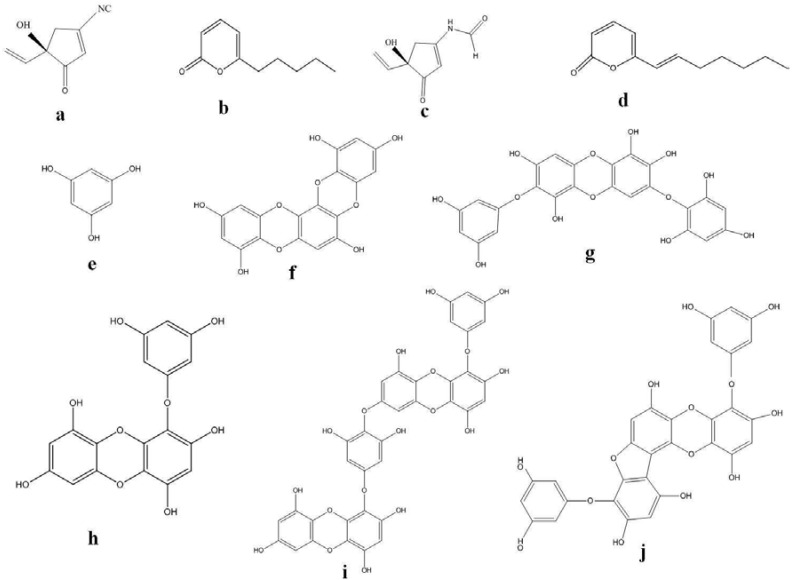
Structures of tyrosinase inhibitors from marine sources.

Skin whitening has been in practice around the world, with Asia as its largest market. Tyrosinase inhibitors (Figure 1) are the most common approach to achieve skin hypo-pigmentation, as this enzyme catalyzes the rate-limiting step of pigmentation. Despite the large number of tyrosinase inhibitors *in vitro*, only a few are able to show induced effects in clinical trials. We review some potential marine organisms with their effects on the pigmentation of skin, focusing mainly on tyrosinase inhibitors. Hence, development of novel tyrosinase inhibitors from natural resources continues to arouse great attention, and in recent years, marine algae have attracted great attention in the search of natural tyrosinase inhibitor agents [56].

**Figure 2:**
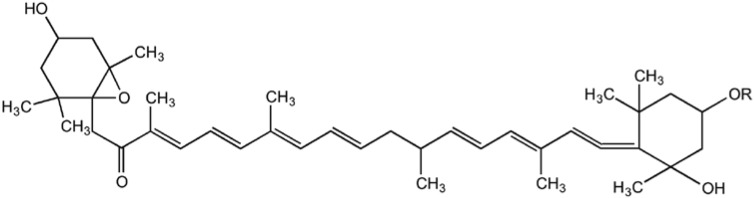
Structure of fucoxanthin.

Fucoxanthin (Figure 2) isolated from *Laminaria japonica* has been reported to suppress tyrosinase activity in UVB-irradiated guinea pig and melanogenesis in UVB-irradiated mice. Oral treatment of fucoxanthin significantly suppressed skin mRNA expression related to melanogenesis, suggesting that fucoxanthin negatively regulated melanogenesis factor at transcriptional level [57]. 

Phloroglucinol derivatives, a common secondary metabolite constituent of brown algae, possess tyrosinase inhibitory activity due to their ability to chelate copper in this enzyme [58]. The effectiveness of brown algal polyphenols in inhibiting UVB-induced skin carcinogenesis in an SKH-1 hairless mouse skin model was investigated by Hwang *et al.* [51]. These *in vivo* reports demonstrated that both dietary feeding and topical treatment of brown algal polyphenols has suppressed cyclooxygenase-2 (COX-2) expression and cell proliferation. These results suggest the role of brown algae polyphenols, phlorotannins, as potential cancer chemopreventive agents against photocarcinogenesis and other adverse effects of UVB exposure. The following table depicts phloroglucinol derivatives from marine algae and their potential skin protective effects (Table 1). These evidences suggest that bioactive compounds derived from marine algae have a promising potential to be used as skin whitening agents.

**Table 1:** Phloroglucinol derivatives and their hypoallergenic effects.

Serial Number	Medicinal Application	Phlorotannin and Brown Algal Species	References
1	Inhibitory effect on histamine release	6,6′-bieckol— *E. cava*	[59]
		Methanolic Extracts— *E.arborea*	[60]
		Eckol	[61]
		6,6′-bieckol	
		6,8′-bieckol	
		8,8′-bieckol— *E.arborea*	
		Phlorofucofuroeckol A	
		Phlorofucofuroeckol B	
2	Inhibitory effect hyaluronidase	Eckol	[62]
		Phlorofucofuroeckol A— *E. bicyclis*	
		Dieckol *E. kurome*	
		8,8′-bieckol	
3	Inhibitory effect on FcεRI overexpression	Methanolic Extracts— *E. cava*	[63]
4	Inhibitory effect on overexpression of IgE	Phlorofucofuroeckol A— *E.arborea*	[30,61]
5	Inhibitory effect of MMP-1 expression	Eckol	[64]
		Dieckol	

**Figure 3:**
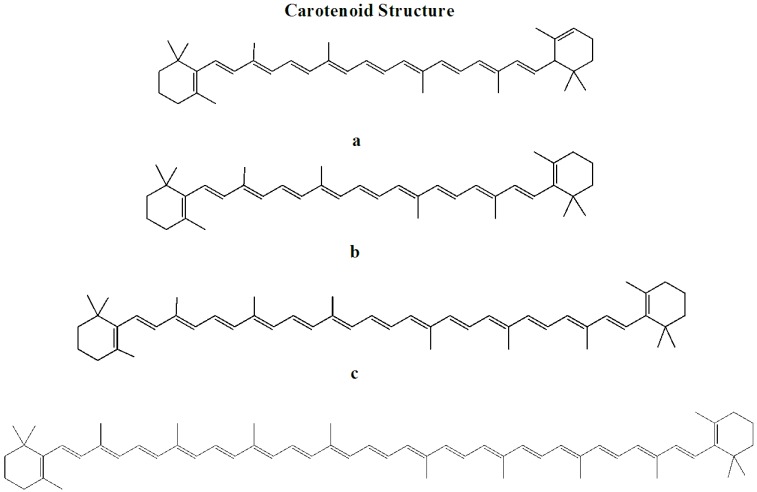
Structure of carotenoids.

**Figure 4:**
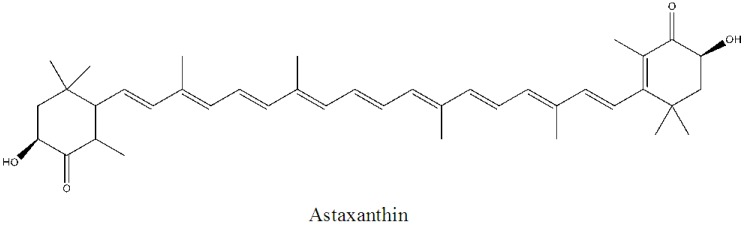
Structure of astaxanthin.

Marine algae-derived carotenoids (Figure 3) and astaxanthin (Figure 4) have been explored for cosmeceutical purposes [19,65,66,67,68,69,70,71]. 

### 2.2. Prevention of Skin-Related Protozoan Diseases

The encounter between humans and infectious agents has been recognized since ancient times. There are various types of infectious agents, such as bacteria, viruses, and fungi, which cause various types of diseases in humans, and the outcome of the disease symptoms is contingent upon the disease-causing agents [72]. Humans have produced various types of treatments/remedies for different types of bacterial diseases since ancient times by using a variety of practices, such as Ayurveda, depending on the availability of the natural resources in those countries [73]. 

Bioactive sesquiterpenes isolated from red algae species *Laurencia rigida*; *Laurencia luzonesis* yielded deschloroelatol, elatol, luzonenone, luzofuran, 3,4-epoxypalisadin, 1,2-dehydro-3,4-epoxypalisadin B, and 15-hydroxypalisadin; and a new diterpene former has shown antibacterial action on *Bacillus megaterium*, and also possesses antifungal action [74]. Crude extracts, purified diverse phlorotannins extracted from brown algae, *Ecklonia kurome* tested on multiresistant *Staphylococcus aureus* and food-borne pathogens, exhibited the antibacterial activity on Gram-positive bacteria, *S. aureus*, and *B. cereus*, and Gram-negative bacteria, *C. jejuni*, *E. coli*, *S. Enteritidis*, *S. typhimurium*, and *V. parahaemolyticus* [75]. Diethyl ether extracts of seaweeds *Cystoseira mediterranea*, *Enteromorpha linza*, *Ulva rigida*, *Gracilaria gracilis* and *Ectocarpus siliculosus* are isolated from the Urla coast (Turkey showed effective results against all test organisms such as *Candida* sp., *Enterococcus faecalis*, *S. aureus*, *Streptococcus epidermidis*, *Pseudomonas aeruginosa* and *Escherichia coli*). Fresh weights of algae extracted using the diethyl ether showed the strong broad spectrum antibiotic activity against the tested bacterial strains; moreover, they have shown more activity against the Gram-positive bacteria, which was more when compared to the Gram-negative bacteria [76]. The latest improvements in science and technology explored the untapped potentials of marine resources.

### 2.3.Atopic Dermatitis

Atopic dermatitis (AD) is a pruritic inflammatory skin disorder associated with a personal or family history of allergy. AD can occur at any age; most often, it affects infants and young children. In some instances, it may persist into adulthood or actually appear only later in life [77,78,79,80,81]. The prevalence of AD is on the rise and is estimated at ~17% in the USA. The fundamental lesion in AD is a defective skin barrier that results in dry itchy skin, and is aggravated by mechanical injury inflicted by scratching. This allows entry of antigens via the skin and creates a milieu that shapes the immune response to these antigens [82]. Clinical observations suggest that AD is the cutaneous manifestation of a systemic disorder that would give rise to asthma, food allergies, and allergic rhinitis [83]. 

Recent studies reveal that the coupling of the IgE onto the Fcε receptors without the help of antigens could elicit such inflammatory responses [84]. Thus it is evident that inhibition of IgE production or reduction in the concentration of IgE would be the best therapeutic approach in treating atopic diseases like AD which is one of the most challenging skin inflammatory diseases. In addition, most recently, a recombinant humanized anti-IgE monoclonal antibody (mAb), omalizumab, used to treat severe asthma, has shown promising effects in the treatment of few cases of AD [85]. However, the challenging aspect with this treatment is an enormous amount of very expensive mAb is required to remove IgE from patients with AD, since the level of serum IgE in many patients with AD is ten to a hundred times higher than those with asthma or allergic rhinitis. 

Marine brown algae-derived phlorotannins have been investigated for their human beneficial aspects that include anti-inflammatory, and hyaluronidase inhibitory activities. *In vitro* studies with the methanol extracts from marine brown algae *Eisenia arborea* have shown 74.3 ± 33.8% inhibition of histamine release from rat basophile leukemia cells (RBL-2H3) sensitized with antidinitrophenyl (DNP) IgE and stimulated with DNP-BSA. These observations suggest that methanol extract which is rich in phlorotannins exhibit the potentiality to treat histamine-related inflammatory diseases that include AD [60]. Hyaluronidase (HAase, EC.3.2.1.35) is an enzyme that depolymerizes the polysaccharide hyaluronic acid (HA) in the extracellular matrix of connective tissue, and is known to be involved in allergic effects [62,86,87,88]. Six phlorotannins: phloroglucinol, an unknown tetramer, eckol (a trimer), phlorofucofuroeckol A (a pentamer), dieckol and 8,8′-bieckol (hexamers), obtained from brown algae *Eisenia bicyclis* and *Ecklonia kurome* were tested *in vitro* for their ability to inhibit hyaluronidase activity. It was reported that these crude phlorotannins had a stronger inhibitory effect on hyaluronidase than well-known inhibitors such as catechins and sodium cromoglycate. According to these findings, 8,8′-bieckol has shown stronger hyaluronidase inhibition with an IC_50_ value of 40 μm, which was about seven times stronger than that of DSCG (a major and active component of anti-allergic drugs) [62]. This thus suggests the importance of polyphenolic derivatives from marine algae as potential anti-inflammatory substances that could be useful leads for cosmeceuticals to treat AD.

Due to the abundant content of phloroglucinol derivatives in *Ecklonia cava*, it is used as a food ingredient and folk medicine against allergic diseases in Asian countries, specifically Korea. Crude extract from *E. cava* was investigated for its anti-allergic activity by Le *et al*. [59]. Their chemical investigation led to the isolation of the two main bioactive phlorotannin derivatives 6,6′-bieckol and 1-(3′,5′-dihydroxyphenoxy)-7-(2″,4″,6-trihydroxyphenoxy)-2,4,9-trihydroxydibenzo-1,4-dioxin for the first time from this genus, together with phloroglucinol and dieckol. These derivatives were assessed by a histamine release assay on human basophilic leukemia (KU812) and rat basophilic leukemia (RBL-2H3) cultured cell lines, respectively. Furthermore, flow cytometric analysis indicated that the potential anti-allergic mechanism is due to the suppression of binding activity between IgE and FcεRI [60]. 

In order to understand the cellular and molecular histopathological mechanisms and the expression pattern of chemokines in AD, *Dermatophagoides farinae* extracts (DfE)-induced NC/Nga AD model are developed in mice and are considered as indispensable animal models to derive a proper medicinal approach to treat this skin inflammation. Recently, the effect of methanolic extracts of *Ecklonia cava* (MEEC) on *Dermatophagoides farina* antigen-induced NC/Nga mouse model have been evaluated by a research group at Biotoxtech, Korea. The MEEC are considered to be rich in polyphenolic components, especially phlorotannins. These phlorotannins from marine algae are reported to exhibit anti-inflammatory activities by several research groups. The *Dermatophagoides farina* antigen-induced NC/Nga mice were administered with MEEC (subcutaneous injection) at concentrations of 3.3 mg/mL, 10 mg/mL and 30 mg/mL. These mice have shown a remarkable recovery after 21 days of administration of MEEC, and the results are more promising when compared to the commercial drug Betamethasone [89]. These findings confirm the effectiveness of polyphenolic compounds as potential cosmeceutical leads for the formulations of lotions and creams to cure AD. 

**Figure 5:**
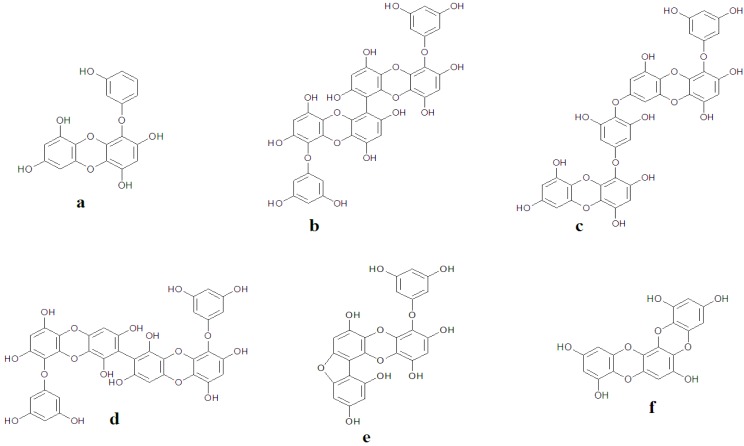
Chemical structures of some phlorotannins: (**a**) Eckol; (**b**) 6,6′-Bieckol; (**c**) Dieckol; (**d**) 8,8′-Bieckol; (**e**) Fucofuroeckol-A; (**f**) Dioxynodehydroeckol.

**Figure 6:**
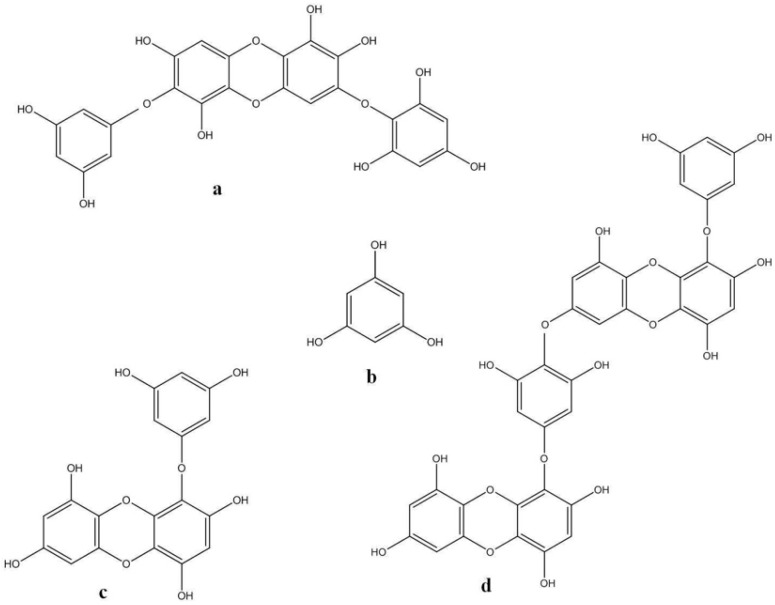
Chemical structures of some phlorotannins: (**a**) diphlorethohydroxycarmalol; (**b**) phloroglucinol; (**c**) eckol; (**d**) dieckol.

A new phlorotannin, phlorofucofuroeckol-B, was isolated from *Eisenia arborea*, an edible brown algae that is occasionally used as a folk medicine in gynecopathy in Japan. The *in vitro* studies on rat basophile leukemia (RBL)-2H3 cells confirmed that this phlorotannin is capable of inhibiting histamine release assuring the anti-allergic property [90]. Eckol, 6,6′-bieckol, 6,8′-bieckol, 8,8′-bieckol, phlorofucofuroeckol-A, and phlorofucofuroeckol-B obtained from *Eisenia arborea* have been reported to exhibit activities (Figure 5 and Figure 6) similar to, or greater than, the typical inhibitor for allergies, epigallocatechin gallate. Phlorofucofuroeckol-B showed the greatest activity among the tested phlorotannins at 2.8-times greater than epigallocatechin gallate [61], thereby suggesting the need for more advanced scientific investigations and animal model studies to unravel the molecular mechanism of phlorotannins as anti-inflammatory substances.

The extract was also capable of reducing the binding between IgE or serum IgE and cell surface FcεRI. RT-PCR analysis revealed that EC extract reduced the mRNA expression of total cellular FcεRI α-chain. Fluorescence spectrophotometry studies showed that the extract inhibited the FcεRI-mediated release of histamine in a concentration-dependent manner. Therefore, these results suggest that EC extract might exert its anti-allergic activity through the negative-regulation of FcεRI expression and a decrease in histamine release [63]. 

On the other hand, the effects of 80% methanol extracts from frozen samples of 41 macroalgae and one sea grass collected in the Ise-Shima region of Japan were investigated on histamine release from rat basophile leukemia cells (RBL-2H3) sensitized with antidinitrophenyl (DNP) IgE and stimulated with DNP-BSA. Of the 21 brown, 5 green, 15 red algae, in addition to the one sea grass tested, extracts only from seven brown algae showed histamine release inhibitory activity from RBL cells [60]. According to these investigations, it is understood that phloroglucinol derivatives possesses the ability to reduce the production of IgE and other inflammatory responses. 

Moreover there are several reports confirming the ability of skin disease treatment abilities of phlorotannins. For example, the phlorotannins eckol and dieckol isolated from *E. stolonifera* have attenuated the expression of MMP-1 expression in human dermal fibroblasts. These findings reveal that the inhibition of MMP-1 (which is an interstitial collagenase, is mainly responsible for the degradation of dermal collagen in human skin aging process) expression by *E. stolonifera* derived phlorotannins was in correlation with the inhibition of both NF-κB and activator protein-1 (AP-1) reporter activity [64]. This unique feature of phlorotannins in repairing skin damages from various allergens could be exploited for the better treatment of ever-challenging AD. More studies have to be focused in the screening of novel compounds from marine algae that could find themselves a prominent place in the treatment of not only AD, but also various other skin inflammations. In addition, as the marine environment includes sponges, molluscs, bryozoans, coelenterates, echinoderms, tunicates, and other marine microorganisms, and reports suggest that most of them do possess anti-inflammatory substances within themselves, a wide choice of cosmeceutical compounds that could cure AD are proposed to researchers.

### 2.4. The Role of Fucoidan in Skin Diseases Treatment

**Figure 7:**
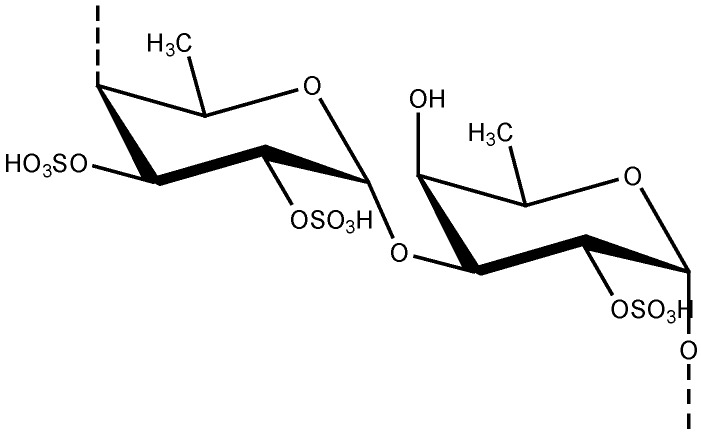
Chemical structure of fucoidan unit.

Marine macroalgae are considered as dietary components and also as alternative medicine in Asian countries like Japan, Korea and China [20]. Marine algae are reported to produce different polysaccharides, including alginates, laminarans, and fucoidans. They usually contain large proportions of L-fucose and sulfate, together with minor amounts of other sugars such as xylose, galactose, mannose, and glucuronic acid [91]. Especially fucoidans from marine algae have been reported to exhibit outstanding biological activities that aid human health [92]. Fucoidans are sulfated polysaccharides that are exclusively found in seaweeds in their cell walls (Figure 7) [93]. This polysaccharide ingredient is composed a polymer of α1→3-linked 1-fucose with sulfate groups on some of the fucose residues at the 4 positions [94].

Fucoidan is being studied extensively due to potential antitumor, antiviral, anticomplement and anti-inflammatory activities [95]. In the skin-related diseases, UV-B reduces type I procollagen levels and increases MMP-1 levels in human skin and plays a major role in the process of photoaging [96]. Fucoidan inhibits UVB-induced MMP-I expression at the protein and mRNA levels in human skin fibroblasts (HS68). Fucoidan treatment also increased type I procollagen mRNA and protein expression in a dose-dependent manner compared to the control. Our data indicate that fucoidan may prevent UVB-induced MMP-I expression and inhibit downregulation of type I procollagen synthesis. We suggest that fucoidan may be a potential therapeutic agent to prevent and treat skin photoaging [97].

Brown algae-derived fucoidan has been reported to show strong inhibition ability on UVB-induced MMP-1 expression *in* *vitro*. In an investigation by Moon *et al.* [41], human skin fibroblast (HS68) cells were pretreated by various concentrations of fucoidan and then subjected to UV-B irradiation (100 mJ/cm^2^). It is known that ultraviolet (UV) B irradiation induces the production of MMPs by activating cellular signaling transduction pathways, which are responsible for the degradation or synthesis inhibition of collagenous extracellular matrix in connective tissues, causing skin photoaging. Their results have suggested that fucoidan from algae has successfully inhibited the expression of MMP-1 by the suppression of extracellular signal regulated kinase (ERK). Moreover, in fucoidan treated cells, the expression of MMP-1 mRNA has been significantly reduced [98]. As brown edible algae are considered dietary food stuff, the consumption of brown algae rich in fucoidan could be beneficial in reducing the risk of MMP-related diseases. 

**Figure 8:**
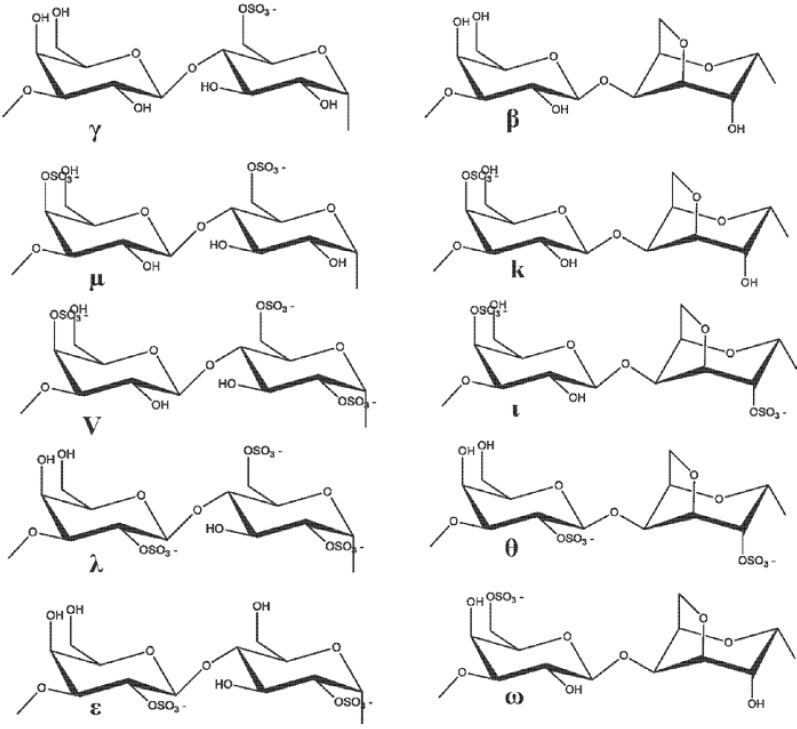
Structure of carrageenan.

Similarly, another research group reported the MMP inhibitory effect of a 16 kDa fucoidan fraction from seaweeds on the parameters involved in the connective tissue breakdown. It was observed that this 16 kDa fucoidan was able to successfully inhibit the gelatinase with a secretion and stromelysin 1 induction by interleukin-1β on dermal fibroblasts *in vitro*. In addition, *ex vivo* studies using the tissue sections of human skin have revealed that this polysaccharide was able to minimize human leukocyte elastase activity, resulting in the protection of human skin elastic fiber network against the enzymatic proteolysis due to this serine proteinase [99]. These findings clearly suggest the potential role of seaweed fucoidans in reducing the risk of some inflammatory pathologies that involves extracellular matrix degradation by MMPs. Usually, high molecular weight (HMW) fucoidans are known to bind growth factors, such as fibroblast growth factor (FGFs), and protect them from proteolysis [100]. The therapeutic ability of fucoidans is thought to be that they can release the glycosaminoglycan-bound stromal-derived factor-1 (SDF-1) from its tissue storage sites. SDF-1 mobilizes medullary progenitors which could participate in angiogenesis with vascular endothelial growth factor and FGF [101,102]. A fraction of low-molecular-weight fucoidan (7 ± 2 kDa) obtained by radical depolymerization of HMW extracts from brown seaweed have been reported to promote therapeutic revascularization in a rat model of critical hind limb ischemia [103]. Normally, MMP-9 plays an important role in both animal models of cerebral ischemia and human stroke. The expression of MMP-9 is elevated after cerebral ischemia, which is involved in accelerating matrix degradation, disrupting the blood–brain barrier, increasing the infarct size, and relating to hemorrhagic transformation [104]. The therapeutic ability of seaweed fucoidans would be a best option in managing the MMP-associated cerebral ischemia. Carrageenan (Figure 8) is also one of the most extensively studied sulfated polysaccharides from marine algae for cosmeceuticals. 

## 3. The Function of MMP Inhibitors in Skin-Related Diseases

**Figure 9:**
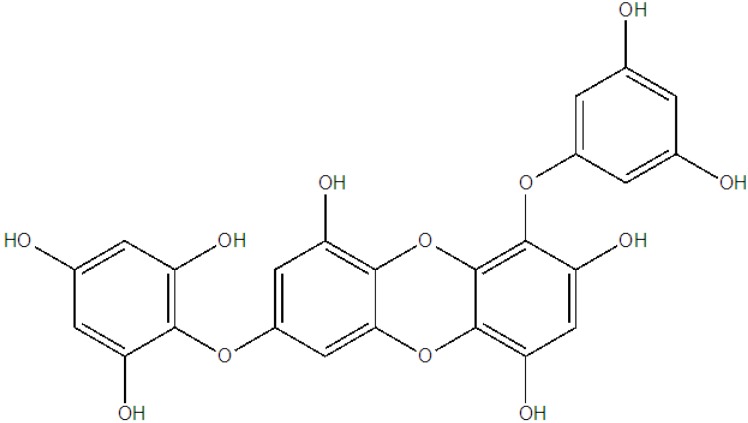
Chemical structure of 1-(3′,5′-dihydroxyphenoxy)-7-(2″,4″,6-trihydroxy-phenoxy)-2,4,9-trihydroxydibenzo-1,4-dioxin.

We have already extensively discussed the MMP inhibitor’s role in skin-related diseases. The MMPs are categorized into three major functional groups. The main three groups include interstitial collagenases that have affinities toward collagen types I, II, and III, (MMP-1, -8, and -13), the stromelysins with specificity for laminin, fibronectin, and proteoglycans (MMP-3, -10, and -11), and the gelatinases that effectively cleave type IV and V collagen (MMP-2 and -9) [105]. Two phlorotannins, namely dieckol and 1-(3′,5′-dihydroxyphenoxy)-7-(2′,4′,6′-trihydroxyphenoxy) 2,4,9-trihydroxydibenzo-1,4,-dioxin (Figure 9), isolated from the methanol extract of the marine brown alga, *Ecklonia cava*, have been reported to suppress both the protein and gene expression levels of MMP-1, MMP-3, and MMP-13 in human osteosarcoma cells (MG-63). 

**Figure 10:**
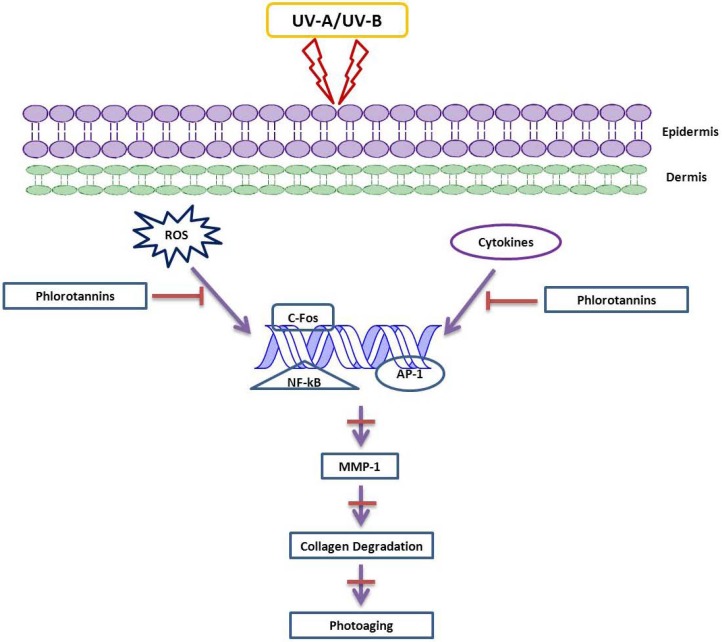
Anti-inflammatory effects of phlorotannins via cytokine blockade.

The inhibitory mechanism effect of phlorotannins against photoaging is shown in Figure 10. This *in vitro* study also reports that these phlorotannins were able to promote osteosarcoma differentiation by collagen synthesis [106]. Similarly, dieckol and eckol isolated from *Ecklonia stolonifera* have inhibited the expression of MMP-1 in human dermal fibroblast cell, *in vitro* [64]. More precisely this investigation suggested that these phlorotannins interfere with the expressions of NF-κB and activator protein-1 (AP-1) which, in turn, enhances the MMP-1 expression that leads to skin-related damages [107]. Hence, it can be recommended that brown algae be recommended as food with medicinal value that can also support skin care. 

## 4. Future Prospects

Furthermore, until now, the majority of the phlorotannins and sulfated polysaccharides reported were from the members of the species *Ecklonia* and *Eisenia*. Many more brown algal members have to be screened for novel phlorotannins and polysaccharide derivatives that can be recommended as potential MMPIs. AD is one the most challenging skin diseases that is still in need of effective and efficient lead compounds for a complete cure. Several topical formulations are already available for the management of this skin disease. However, the permanent cure for this disease is still not completely achieved. In addition, health risks and seafood allergies should also be considered in the further cosmeceutical development of such a product. 

## 5. Conclusions

The effectiveness of marine algal compounds in proper downregulation of MMPs, tyrosinase inhibitor activity and related pathological (bacteria and fungi) effects has been explored thoroughly in the present review. In conclusion of this review, brown algae-derived phlorotannins and sulfated polysaccharides will be playing a major role in the cosmeceutical production development of the future. The proper development and isolation of bioactive compounds from algae will be undoubtedly helpful in cosmeceutical product development. 
